# Diabetes medications and associations with Covid-19 outcomes in the N3C database: A national retrospective cohort study

**DOI:** 10.1371/journal.pone.0271574

**Published:** 2022-11-17

**Authors:** Carolyn T. Bramante, Steven G. Johnson, Victor Garcia, Michael D. Evans, Jeremy Harper, Kenneth J. Wilkins, Jared D. Huling, Hemalkumar Mehta, Caleb Alexander, Jena Tronieri, Stephenie Hong, Anna Kahkoska, Joy Alamgir, Farrukh Koraishy, Katrina Hartman, Kaifeng Yang, Trine Abrahamsen, Til Stürmer, John B. Buse, N3C core authors

**Affiliations:** 1 Division of General Internal Medicine, Department of Medicine, University of Minnesota Medical School, Minneapolis, Minnesota, United States of America; 2 Institute for Health Informatics, University of Minnesota Medical School, Minneapolis, Minnesota, United States of America; 3 Department of Biomedical Informatics, Stony Brook University Hospital, Stony Brook, New York, United States of America; 4 Biostatistical Design and Analysis Center, University of Minnesota Medical School, Minneapolis, Minnesota, United States of America; 5 Owl HealthWorks, Indianapolis, IN, United States of America; 6 Biostatistics Program, Office of the Director, National Institute of Diabetes and Digestive and Kidney Disease, Bethesda, Maryland, United States of America; 7 Division of Biostatistics, University of Minnesota School of Public Health, Minneapolis, Minnesota, United States of America; 8 Division of Epidemiology and Methodology, Johns Hopkins School of Public Health, Baltimore, Maryland, United States of America; 9 Division of General Internal Medicine, Department of Medicine, Johns Hopkins School of Medicine, Baltimore, Maryland, United States of America; 10 Department of Psychiatry, Perelman School of Medicine at the University of Pennsylvania, Philadelphia, Pennsylvania, United States of America; 11 Department of Nutrition, Gillings School of Global Public Health, University of North Carolina at Chapel Hill, Chapel Hill, North Carolina, United States of America; 12 ARIScience, Boston, Massachusetts, United States of America; 13 Division of Nephrology, Stony Brook University Hospital, Stony Brook, New York, United States of America; 14 Novo Nordisk, Bagsvaerd, Denmark; 15 Department of Epidemiology, Gillings School of Global Public Health, University of North Carolina at Chapel Hill, Chapel Hill, North Carolina, United States of America; 16 Division of Endocrinology, Department of Medicine, University of North Carolina Medical School, Chapel Hill, North Carolina, United States of America; University of Phayao, THAILAND

## Abstract

**Background:**

While vaccination is the most important way to combat the SARS-CoV-2 pandemic, there may still be a need for early outpatient treatment that is safe, inexpensive, and currently widely available in parts of the world that do not have access to the vaccine. There are in-silico, in-vitro, and in-tissue data suggesting that metformin inhibits the viral life cycle, as well as observational data suggesting that metformin use before infection with SARS-CoV2 is associated with less severe COVID-19. Previous observational analyses from single-center cohorts have been limited by size.

**Methods:**

Conducted a retrospective cohort analysis in adults with type 2 diabetes (T2DM) for associations between metformin use and COVID-19 outcomes with an active comparator design of prevalent users of therapeutically equivalent diabetes monotherapy: metformin versus dipeptidyl-peptidase-4-inhibitors (DPP4i) and sulfonylureas (SU). This took place in the National COVID Cohort Collaborative (N3C) longitudinal U.S. cohort of adults with +SARS-CoV-2 result between January 1 2020 to June 1 2021. Findings included hospitalization or ventilation or mortality from COVID-19. Back pain was assessed as a negative control outcome.

**Results:**

6,626 adults with T2DM and +SARS-CoV-2 from 36 sites. Mean age was 60.7 +/- 12.0 years; 48.7% male; 56.7% White, 21.9% Black, 3.5% Asian, and 16.7% Latinx. Mean BMI was 34.1 +/- 7.8kg/m^2^. Overall 14.5% of the sample was hospitalized; 1.5% received mechanical ventilation; and 1.8% died. In adjusted outcomes, compared to DPP4i, metformin had non-significant associations with reduced need for ventilation (RR 0.68, 0.32–1.44), and mortality (RR 0.82, 0.41–1.64). Compared to SU, metformin was associated with a lower risk of ventilation (RR 0.5, 95% CI 0.28–0.98, p = 0.044) and mortality (RR 0.56, 95%CI 0.33–0.97, p = 0.037). There was no difference in unadjusted or adjusted results of the negative control.

**Conclusions:**

There were clinically significant associations between metformin use and less severe COVID-19 compared to SU, but not compared to DPP4i. New-user studies and randomized trials are needed to assess early outpatient treatment and post-exposure prophylaxis with therapeutics that are safe in adults, children, pregnancy and available worldwide.

## Introduction

The novel severe acute respiratory syndrome coronavirus 2 (SARS-CoV-2) continues to spread globally and evolve into variants that may be more infectious and may evade current vaccines and therapies [1]. While vaccine development and distribution remains the primary way to combat the COVID-19 pandemic, many individuals around the world do not yet have access to these vaccines, young children are not yet vaccinated, and large percentages of those with access are not willing to be vaccinated [2, 3]. Thus there appears to be a need for early outpatient treatment options that are safe, inexpensive, and widely available to prevent severe symptoms, hospitalization, critical illness, and mortality associated with SARS-CoV-2 infection.

To this end, several medications have been suggested for repurposing to treatment of SARS-CoV-2 [4]. Of these, metformin seemed to warrant further investigation given its widespread use in adults, children, pregnancy, and its availability worldwide for less than $2 per month [5–9]. Metformin is known to inhibit mTOR (mechanistic target of rapamycin), which appears to be important for replication of SARS-CoV-2 [10, 11]. Metformin has been shown to inhibit the viral life cycle of other RNA viruses [12]. Beyond affecting the viral life cycle, metformin has anti-inflammatory and anti-thrombotic properties, which may also reduce severity of COVID-19 disease [13–15]. In addition, there are in-vitro, in-silico, and observational data suggesting that metformin use may reduce the severity of COVID-19 disease [10, 11, 16–18]. However, observational analyses are limited because of confounding by indication, which is particularly relevant for metformin because diabetes is a risk factor for poor outcomes from COVID-19. One pharmaco-epidemiologic approach to assess potential pleiotropic effects of medications while minimizing confounding by indication is to compare individuals with the same condition (the same indication), and same engagement in healthcare (taking similarly-available medications), on therapeutically equivalent medications.

From a type 2 diabetes (T2DM) treatment standpoint, metformin, Dipeptidyl peptidase 4 inhibitors (DPP4i), and sulfonylureas (SU’s) are therapeutically equivalent. Thus, comparing individuals with type 2 diabetes treated with monotherapy of one of these three medications may reduce confounding by indication, which is important given that diabetes is a significant risk factor for poor outcomes from COVID-19. DPP4i have been hypothesized to reduce severity of COVID-19 disease, by reducing viral entry into the cell and DPP4i’s have also been associated with reduced inflammation, and blocking viral entry has not been a strong pathway for stopping the virus in other medications [19–21]. Metformin has more favorable profile regarding cost and medication interactions, so it is important to understand whether it would offer benefit compared to DPP4i’s. Sulfonylureas have no hypothesized benefit in SARS-CoV-2 infection beyond treatment of pre-existing diabetes in individuals with comorbidities. Comparing metformin to SU’s is important for understanding if metformin offers any benefit beyond treating diabetes.

Previous observational analyses assessing metformin and COVID-19 outcomes have had limitations such as geographic homogeneity, lack of BMI data, and insufficient numbers for comparing diabetes monotherapy groups [17, 18, 22–24]. Our objective was to address some of these limitations by using the N3C database (National COVID Cohort Collaborative), a large nationally representative dataset of electronic health record (EHR) data, [25] to assess COVID-19 outcomes in adults with type 2 diabetes (T2DM). We used an active comparator design of prevalent users of diabetes monotherapy: metformin versus sulfonylureas (SU) and DPP4i. We hypothesized that metformin use prior to SARS-CoV-2 infection would be associated with less severe COVID-19 outcomes than SU and DPP4i use.

## Methods

### Design and population

We performed a retrospective cohort analysis of patient-level, de-identified EHR data from 2017 to May 2021. The N3C includes data from 56 institutions nationally, across geographically and diverse areas [25]. This analysis was approved by the University of Minnesota institutional review board (STUDY00011578), which provided a waiver of consent. We used an active comparator prevalent user design of diabetes monotherapy with either metformin, SU, or DPP4i.

### Inclusion and exclusion criteria

**Fig 1:**
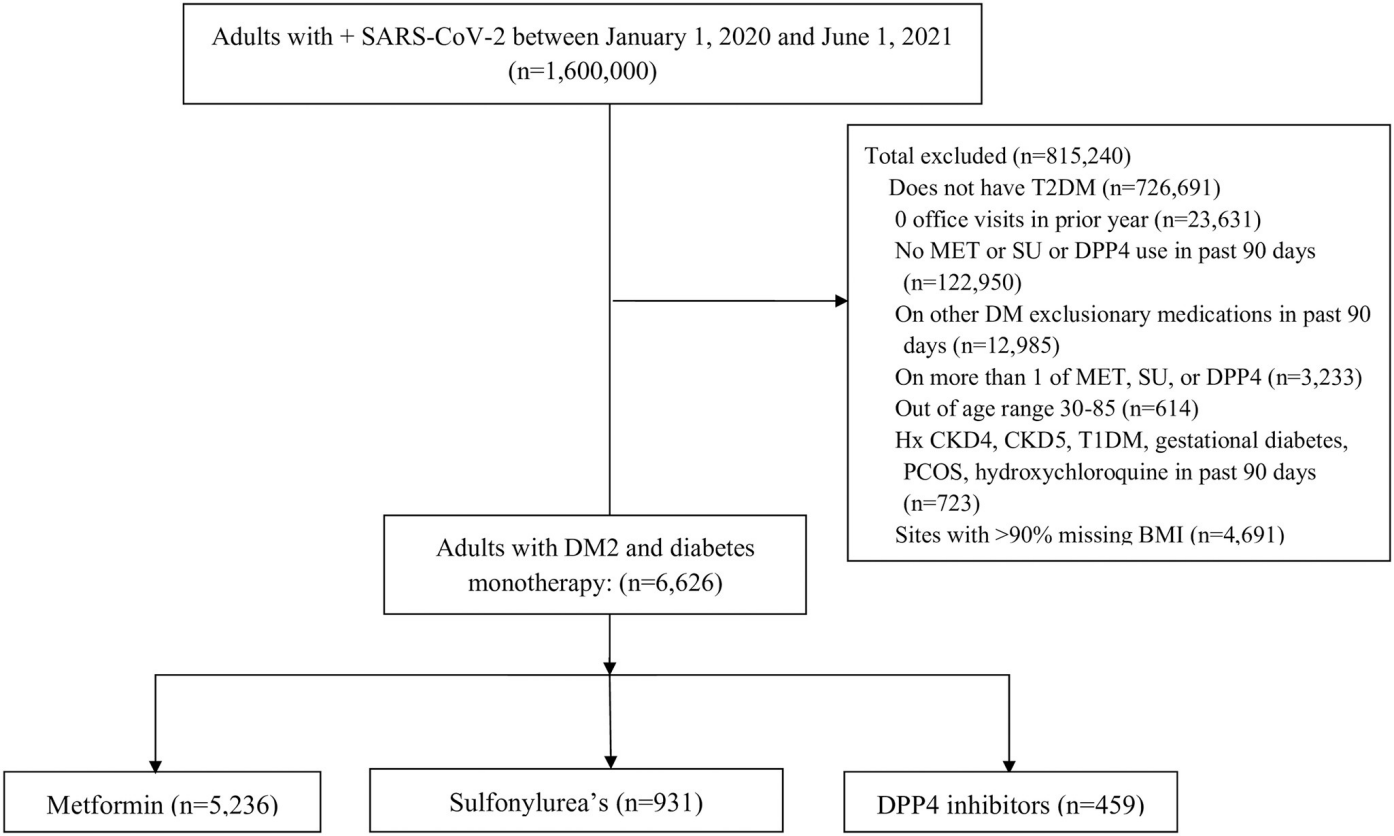
This represents the number of patients included in the analysis after the inclusion and exclusion criteria were applied.

The dataset included 1.6 million individuals with a positive SARS-CoV-2 polymerase chain reaction (PCR) result between 1/1/2020 to 12/12/2020 (Fig 1), with EHR records extending back two years for medical histories. Analysis was restricted to adults over age 30 years with T2DM and at least 1 outpatient healthcare encounter in the 12 months before the +SARS-CoV-2 result. This age minimum was chosen to enrich the population as 30 is the age at which the risk of hospitalization appears to rise above 5% [26]. T2DM was defined as having at least one diabetes pharmacotherapy agent and either a hemoglobin A1C (HbA1C) level > = 6.5% or an ICD-10 code for diabetes in the previous 12 months. To reduce confounding by contraindication, individuals were excluded if they had a diagnosis of chronic kidney disease (CKD) Stage 4, Stage 5, or End Stage Renal Disease (ESRD). Records of individuals with prediabetes or polycystic ovarian syndrome (two common uses for metformin other than T2DM), but not T2DM, were excluded. To reduce confounding by frailty, individuals over age 85 years were excluded.

### Exposure groups

Metformin, SU, and DPP4i use was determined by being reported on the patients’ active medication list within the 90 days prior to the +SARS-CoV-2 result. Using both the WHO ATC classification and RxNorm schemas, concept sets were created for the drugs of interest. A 2-physician review team manually read through each concept expression to assure appropriate inclusion of concepts to the expression list. Individuals were excluded from the analysis if any other diabetes medication were listed in the 90 days prior to the SARS-CoV-2 result.

### Outcomes

The clinical outcomes of interest were hospital admission for COVID-19 disease; need for ventilation for COVID-19 (defined as needing intubation or ECMO); and mortality (in-hospital and before-hospital) from COVID-19 disease. Each outcome was assessed independently, not as a composite outcome [27]. Additionally, back-pain was assessed as a negative control outcome [28]. Back pain was captured using concepts from various vocabularies, including CPT4, HCPCS, ICD10, ICD10CM, SNOMED, and Nebraska Lexicon, to capture outpatient diagnoses related to back pain and its synonyms.

### Covariates

Potentially confounding covariates were identified based on clinical assessment of variables associated with the exposures and outcomes and are included in Table 1. Analysis was also adjusted for site, but this information is not included in Table 1. Comorbidities were defined using translated OMOP concepts from ICD-10 codes in the previous 12 months. For chronic kidney disease, patients were additionally matched on serum creatinine (SCr) within the previous 12 months.

**Table 1:** Demographic and clinical characteristics of adults with Type 2 Diabetes treated by oral monotherapy and SARS-CoV-2 infection.

n (%)		Overall (n = 6,626)	Metformin n = 5,236	DPP4 inhibitors n = 459	Sulfonylureas n = 931	SMD**
Age, mean (SD)		60.69 (11.9)	60.04 (11.9)	62.50 (11.6)	63.43 (11.5)	0.193
Age	30–49	1,225 (18.5)	1,035 (19.8)	60 (13.1)	131 (14.1)	0.213
	50–59	1,732 (26.1)	1,409 (26.9)	121 (26.4)	202 (21.7)	
	60–69	2,039 (30.8)	1,604 (30.6)	142 (30.9)	293 (31.5)	
	70–79	1,289 (19.5)	952 (18.2)	104 (22.7)	233 (25.0)	
	80–85	341 (5.1)	237 (4.5)	32 (7.0)	72 (7.7)	
Male		3,228 (48.7)	2,546 (48.6)	219 (47.7)	463 (49.7)	0.027
Race, Ethnicity	White	3,815 (57.6)	2,988 (57.1)	264 (57.5)	563 (60.5)	0.120
	Black	1,451 (21.9)	1,121 (21.4)	119 (25.9)	211 (22.7)	
	Asian	235 (3.5)	192 (3.7)	<20	27 (2.9)	
	Hispanic/Latinx	1,108 (16.7)	925 (17.7)	58 (12.6)	125 (13.4)	
	Other/Unknown	1,125 (17.0)	935 (17.9)	<60	130 (14.0)	
BMI, mean (SD), kg/m^2^		34.09 (7.80	34.27 (7.85)	33.07 (7.67)	33.58 (7.55)	0.104
BMI Category (kg/m^2)^	< = 25.0	527 (8.0)	389 (7.4)	56 (12.2)	82 (8.8)	0.14
	25.0 < 30.0	1,592 (24.0)	1,232 (23.5)	112 (24.4)	248 (26.6)	
	30.0 < 35.0	1,846 (27.9)	1,475 (28.2)	127 (27.7)	244 (26.2)	
	35.0 < 40.0	1,266 (19.1)	1,012 (19.3)	76 (16.6)	178 (19.1)	
	> = 40.0	1,216 (18.4)	987 (18.9)	74 (16.1)	155 (16.6)	
**Comorbidities, n (%)**						
Heart failure		615 (9.3)	469 (9.0)	50 (10.9)	96 (10.3)	0.043
Coronary Artery Disease		1,103 (16.6)	830 (15.9)	91 (19.8)	182 (19.5)	0.069
Hypertension		5,111(77.1)	3,997 (76.3)	350 (76.3)	764 (82.1)	0.096
COPD		496 (7.5)	369 (7.0)	46 (10.0)	81 (8.7)	0.071
Cancer		755 (11.4)	560 (10.7)	70 (15.3)	125 (13.4)	0.091
Liver disease		205 (3.1)	155 (3.0)	< 20	39 (4.2)	0.067
CKD Stage 1, 2, or 3		794 (12.0)	525 (10.0)	99 (21.6)	170 (18.3)	0.214
Serum creatinine, mean (SD)		0.94 (0.32)	0.92 (0.30)	1.04 (0.40)	1.00 (0.38)	0.233
**Medication prescriptions in the 90 days before +SARS-CoV-2 result**						
ACEi		964 (14.5)	1,091 (20.8)	54 (11.8)	141 (15.1)	0.165
ARB		1,286 (19.4)	794 (15.2)	48 (10.5)	122 (13.1)	0.094
Statins		2,312 (34.9)	1,942 (37.1)	102 (22.2)	268 (28.8)	0.219
Anti-coagulants		605 (9.1)	484 (9.2)	34 (7.4)	87 (9.3)	0.047
Aspirin		579 (8.7)	468 (8.9)	36 (7.8)	75 (8.1)	0.026
**Outcomes from Covid-19**						
Hospitalization, ventilation, or mortality		17.9%	17.0%	22.0%	20.8%	
Back pain (negative control)		1,689 (25.5)	1,331 (25.4)	238 (25.6)	121 (26.4)	

Abbreviations: DPP4i = dipeptidyl peptidase-4 inhibitor; PCR = polymerase chain reaction; SD = standard deviation; BMI = body mass index; SDM = standardized mean difference. COPD = chronic obstructive pulmonary disease; CKD = chronic kidney disease; ACEi = angiotensin converting enzyme inhibitor; ARB = angiotensin receptor blocker;

*p-value for differences between the 3 groups.

**SMD is the average of the 3 pairwise SMD’s.

### Missingness

After excluding sites with greater than 90% missingness for BMI, weight was missing in 6.8%, height was missing in 8.5%, and serum creatinine level was missing in 17.2% of the cohort. With exception of weight, these missing data were addressed using the multiple imputation by chained equations (MICE) algorithm, where each incomplete variable is imputed stochastically by a separate model using fully conditional specification. After using MICE, BMI was missing in 2.7% of the overall cohort. All exposure, outcome, and confounder variables were included in the imputation models. The predictive mean matching method was used, with the passive imputation method used to specify deterministic dependencies among the columns, specifically BMI = weight/height^2^ and the eGFR and creatinine, age, race, and gender relationship specified in the CKD-EPI eGFR equation [29]. Twenty completed data sets were constructed, the exposure and outcome models were fit to each data set separately as described below, and results were pooled using the Rubin method [30].

### Statistical analyses

For descriptive purposes, categorical variables were presented using counts and percentages, and continuous variables presented as means and standard deviation, for each exposure group. Differences among the 3 groups were summarized using the average Standardized Mean Difference (Table 1).

**Fig 2:**
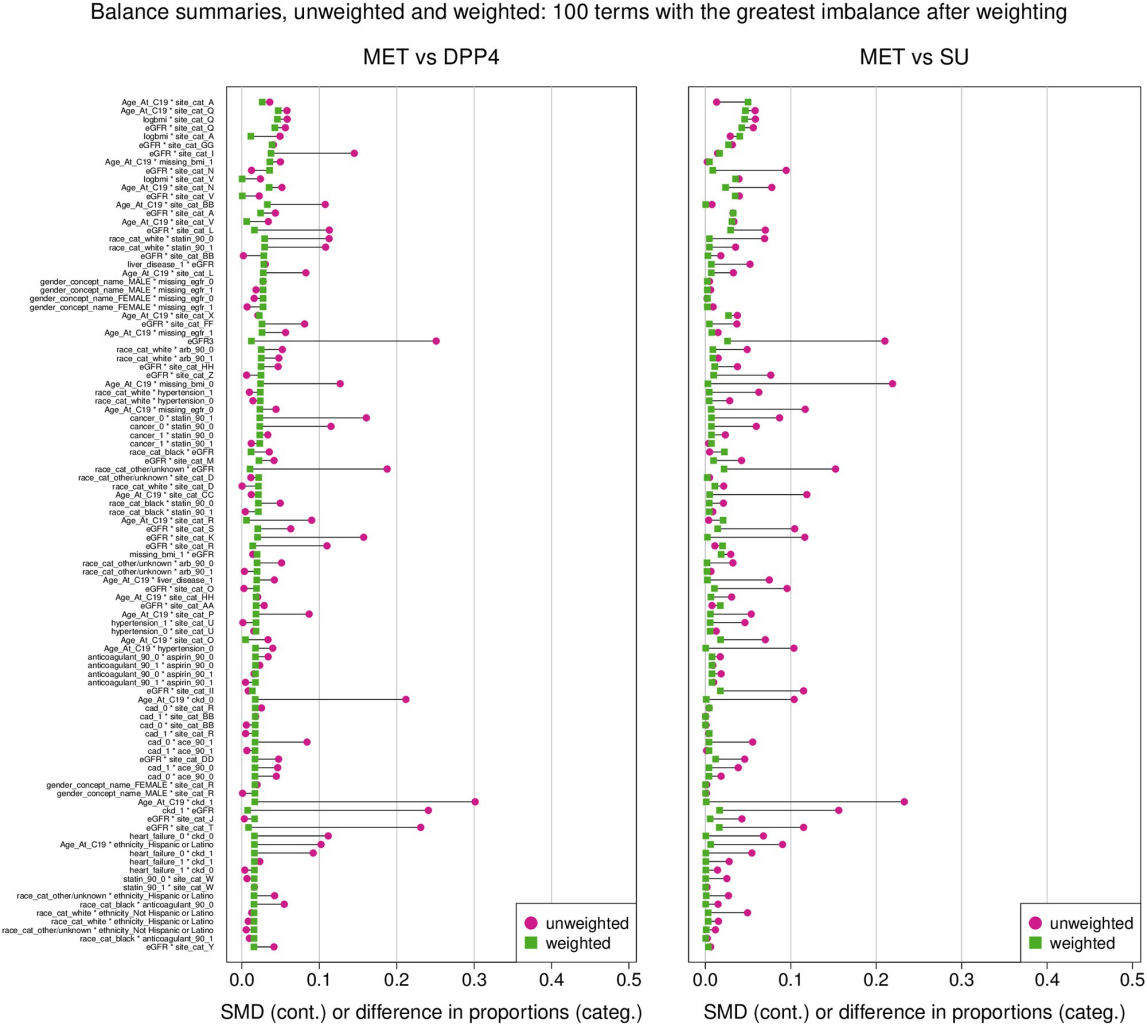
The x axis the standardized mean difference for continuous variables and difference in proportions for categorical variables for the 100 terms with the greatest imbalance after weighing. The circles represent the balance before weighting and the squares represent the balance after weighing.

**Fig 3:**
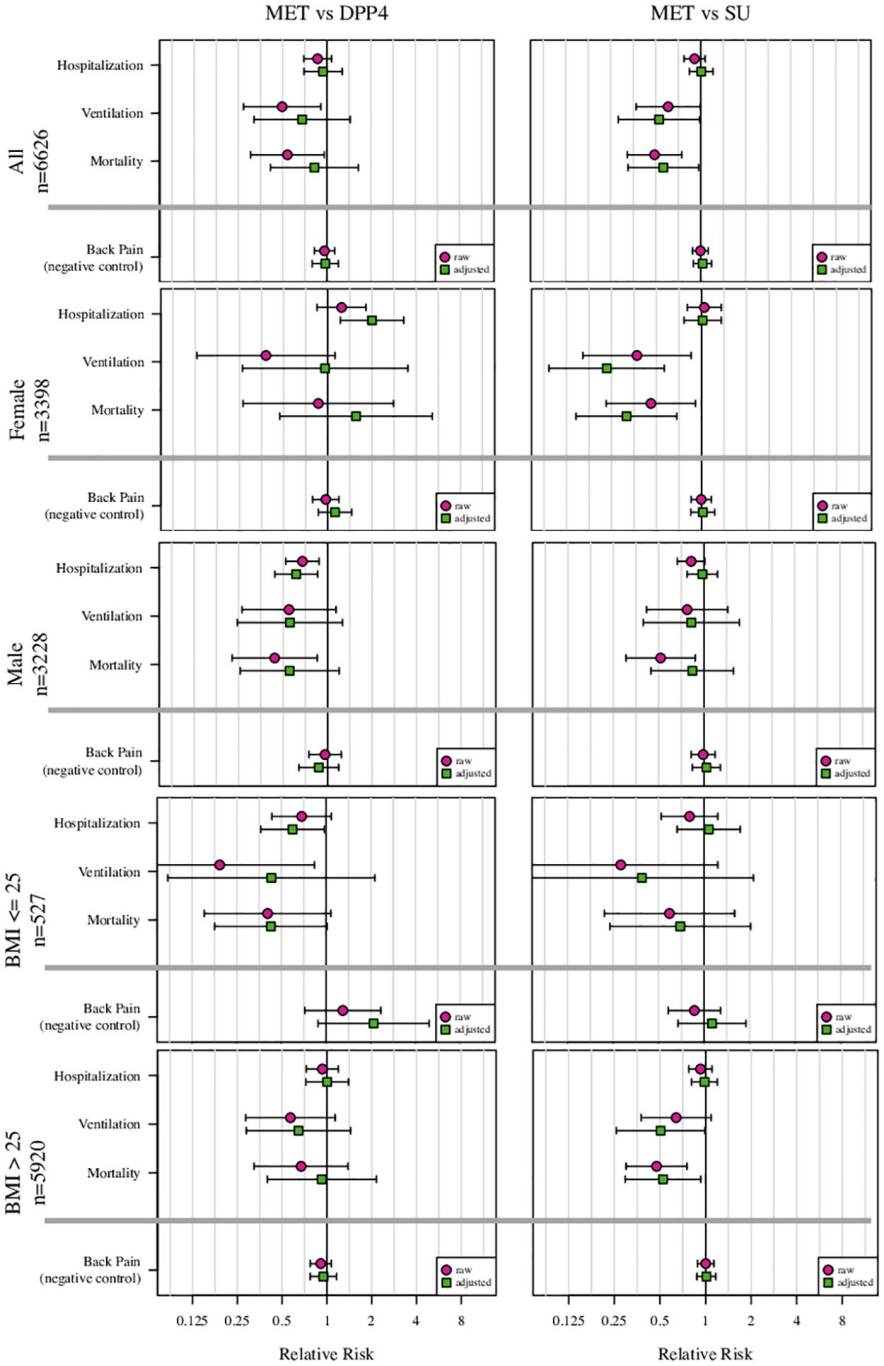
This represents the risk ratios and 95% confidence intervals for the overall cohort as well as for subgroups by gender and subgroups by BMI (<25kg/m^2^ and ≥ 25kg/m^2^). The three panels on the left represent the metformin vs. DPP4i inhibitor comparison. The three panels on the right represent the metformin vs. SU comparison. The circles represent the raw comparison, and the squares represent the adjusted analysis. Within each panel, from top to bottom, the top result is risk of hospitalization; the 2^nd^ result is risk of ventilation (including ECMO); the 3^rd^ result is the risk of mortality; and the 4^th^ comparison is the risk of a back pain, calculated as a negative control outcome. Abbreviations: MET = metformin; DPP4i = dipeptidyl peptidase 4 inhibitors; SU = sulfonylurea.

To adjust for confounding, we estimate weights with entropy balancing [31, 32]. Entropy balancing adjusts for confounding by exactly balancing means of confounders across treatment groups and can be viewed as an indirect approach of estimating the propensity score [33] but is empirically more robust [34]. In the balancing model, we include main effects in log BMI, sex, age, race, ethnicity, site, heart failure, coronary artery disease, chronic obstructive pulmonary disease, cancer, hypertension, liver disease, chronic kidney disease (stage 3 or lower), eGFR, past 90 days use of each of ARB, ACE inhibitor, statin, anticoagulant, and aspirin, and indicators for missing BMI and missing eGFR, and interactions between gender and hypertension, eGFR and statin, eGFR and gender, ACE and sex, eGFR and anticoagulant, eGFR and heart failure, and eGFR and COPD, as we observed substantial imbalances in these interactions. The outcome analysis proceeds in the same manner as an inverse probability of treatment weighting estimate. The summary of the balance between variables can be seen in Fig 2 (the 100 terms with the greatest imbalance before weighting), and Fig 3 (the 100 terms with the greatest imbalance after weighting). After weighting, the standardized absolute mean difference (SMD) was less than 0.05 for all terms.

These weights are then used in fitting a weighted relative risk regression model, which in addition to the balancing weights also includes covariates for all main effects in the balancing model, to construct doubly robust estimates of the relative effects of exposure on each outcome [35].

### Subgroup analyses

Prespecified subgroup analyses were conducted by sex and BMI based on previous literature.

### Sensitivity analyses

In order to understand whether selection bias caused us to misclassify individuals who had these chronic medications prescribed longer than 90 days before their +SARS-CoV-2 infection, we conducted sensitivity analyses using medications defined within the prior 180 and 270 days (S1 Table and S1, S2 Figs in S1 File). To assess for degree of unmeasured confounding that would be necessary to account for observed associations, we calculated e-values using the method outlined by VanderWeele et al [36]. Further sensitivity analyses will soon be possible in the data environment [37].

All analyses were conducted within the secure N3C computing environment using R statistical software (R Foundation for Statistical Computing, Vienna, Austria) including the following packages: mice (for multiple imputation), [38] WeightIt and MatchThem (for estimating balancing weights), [39, 40] cobalt (for summarizing balance statistics), [41] survey (for inverse probability weighted regression), [42] and tableone (for descriptive statistics) [43]. Lastly, the statistical evidence of point/interval estimates were considered prior to relying on p-values’ significance levels [44].

## Results

### Characteristics of the cohort

The total sample included 6,626 adults with T2D and positive SARS-CoV-2 test from 36 sites (Fig 1). The mean age was 60.7 +/- 12.0 years; 48.7% were male; 56.7% were White, 21.9% Black, 3.5% Asian, and 16.7% Latinx. The mean BMI was 34.1 +/- 7.8kg/m^2^. Overall, 14.5% of the sample was hospitalized; 1.5% received mechanical ventilation; and 1.8% died.

The baseline demographic characteristics varied between the monotherapy cohorts: metformin users were younger than the SU and DPP4i users (60.0 versus 63.4 and 62.5 years, respectively). A greater percentage of metformin users were Latinx (17.7%) compared to SU (13.4%) and DPP4i (12.6%). The mean BMI in the metformin group was 34.3kg/m^2^ compared to the SU (33.6) and DPP4i (33.1) groups. The DPP4i and SU groups had higher rates of cardiovascular disease, chronic renal disease, and cancer compared to the metformin group, Table 1.

In unadjusted frequencies, 14.2% of metformin uses were hospitalized, compared to 16.3% and 15.6% of DPP4i and SU users, respectively; 1.3% of metformin users were ventilated, compared to 2.6% and 2.1% of DPP4i and SU users, respectively; and 1.5% of metformin users died from COVID-19, compared to 2.8% and 3.1% of DPP4i and SU users, respectively.

The standardized mean difference between covariates before weighing ranged from approximately 0.05 to 0.50 (Supplement), and after weighting the SMD was < 0.05 for all covariates (Fig 2).

In adjusted outcomes metformin had non-significant associations with reduced severity of COVID-19 compared to DPP4i (Fig 3). Compared to SU, metformin was associated with a lower risk of mortality (RR 0.56, 95%CI 0.33–0.97, p = 0.037) and needing ventilation (RR 0.5, 95% CI 0.28–0.98, p = 0.044). There was no difference between the cohorts in unadjusted or adjusted results of the negative control outcome, back pain (Table 2, Fig 3).

**Table 2:** Risk ratios for severe COVID-19 outcomes in the overall cohort as well as subgroups by gender.

**Overall cohort, metformin versus DPP4i (n = 5,695** * **)**				**Female (n = 2,930** * **)**		**Male (n = 2,756** * **)**	
**Outcome**	**Model**	**RR (95% CI)**	**p value**	**RR (95% CI)**	**p value**	**RR (95% CI)**	**p value**
Hospitalization	Crude	0.87 (0.70–1.08)	0.195	1.24 (0.85–1.82)	0.27	0.61 (0.44–0.86)	<0.01
*Number of events*	Adjusted	0.94 (070–1.27)	0.700	2.00 (1.22–3.29)	<0.01	0.68 (0.52–0.88)	<0.01
	816			373		433	
Ventilation	Crude	0.50 (0.27–0.91)	0.024	0.38 (0.13–1.12)	0.08	0.55 (0.26–1.14)	0.11
	Adjusted	0.68 (0.32–1.44)	0.315	0.96 (0.26–3.50)	0.95	0.56 (0.24–1.26)	0.16
*Number of events*							
	80			21		59	
Mortality	Crude	0.54 (0.30–0.96)	0.036	0.86 (0.26–2.81)	0.81	0.44 (0.23–0.85)	0.02
*Number of events*	Adjusted	0.82 (0.41–1.64)	0.581	1.56 (0.47–5.16)	0.47	0.55 (0.26–1.20)	0.13
	93			32		61	
Back pain	Crude	0.96 (0.82–1.13)	0.656	0.97 (0.79–1.19)	0.77	0.96 (0.75–1.24)	0.76
(negative control)	Adjusted	0.98 (0.79–1.20)	0.816	1.12 (0.86–1.45)	0.84	0.87 (0.64–1.19)	0.39
*Number of events*	1,452			831		621	
**Overall cohort, metformin versus SU (n = 6,167** ** **)**				**Female (n = 3,158** ** **)**		**Male (3,009** ** **)**	
**Outcome**	**Model**	**RR (95% CI)**	**p value**	**RR (95% CI)**	**p value**	**RR (95% CI)**	**p value**
Hospitalization	Crude	0.91 (0.77–1.07)	0.252	1.04 (0.80–1.35)	0.75	0.82 (0.67–1.01)	0.07
*Number of events*	Adjusted	1.01 (0.84–1.21)	0.945	1.02 (0.76–1.35)	0.91	0.98 (0.77–1.23)	0.84
	886			406		480	
Ventilation	Crude	0.60 (037–0.99)	0.046	0.37 (0.16–0.85)	0.02	0.77 (0.42–1.44)	0.42
*Number of events*	Adjusted	0.53 (0.28–0.98)	0.044	0.23 (0.09–0.56)	<0.01	0.82 (0.39–1.72)	0.61
	88			25		63	
Mortality	Crude	0.49 (0.32–0.75)	0.001	0.46 (0.23–0.91)	0.03	0.52 (0.30–0.87)	0.01
*Number of events*	Adjusted	0.56 (0.33–0.97)	0.037	0.31 (0.14–0.68)	<0.01	0.84 (0.44–1.57)	0.58
	109			40		69	
Back pain	Crude	0.99 (0.88–1.12)	0.926	1.00 (0.85–1.16)	0.95	0.99 (0.82–1.19)	0.89
(negative control)	Adjusted	1.03 (0.89–1.18)	0.710	1.02 (0.84–1.22)	0.84	1.04 (0.84–1.29)	0.74
*Number of events*	1,569			894		675	

Abbreviations: DPP4i = dipeptidyl peptidase-4 inhibitors; SU = sulfonylureas.

*n is for metformin + DPP4i users;

**n is for metformin + SU users.

For subgroup analyses, there was evidence that the treatment effect of metformin relative to SU on ventilation differed between females and males with a sex by treatment interaction p = 0.02; and on mortality, p = 0.05 (Table 2, Fig 3). There was no difference in outcomes between BMI subgroups. The sensitivity analyses using 180 and 270 days for capturing chronic medication use showed similar results (Supplement). The e-values for the adjusted model ranged from 1.11 to 8.16. E-values indicate the magnitude of association that an unmeasured confounder would need to have with both the treatment (or in the case of a RR<1 the control, either DPP4i or SU) and outcome, beyond the measured confounders, to account for any observed association.

## Discussion

This analysis of adults with T2DM and +SARS-CoV-2 infection was the first analysis of prevalent users of diabetes monotherapy and was possible because of the size of this database. We found that compared to SU use, metformin use was significantly associated with less severe outcomes from COVID-19 compared to SU users, but associations were not significant compared to DPP4i use. The size of this database allowed us to conduct this analysis with prevalent user comparator groups of diabetes medications that are therapeutically similar, as SU and DPP4i are less common than metformin. We feel this approach has advantages over a non-user comparison, as it explicitly compares to patients receiving an alternative treatment for the same indication, which is a significant consideration when assessing diabetes medications and outcomes from COVID-19 in persons with T2DM. A recent paper by Wang et al, [45] conducted a similar analysis in adults with T2DM comparing metformin to other diabetes medications. They found favorable hazard ratios for metformin compared to the other diabetes medications, but none of the matched analyses reached the 5% level of statistical significance [44].

We conducted a prespecified subgroup analysis by sex based on earlier work showing that metformin lowers CRP more in women than men, improved cancer mortality in women but not men, and conveyed greater protection against severe outcomes from COVID-19 in women compared to men [46]. The association with lower risk of ventilation and mortality with metformin versus SU was significant for females but not for males in this analysis. This potential influence of sex as a biologic variable should be further assessed. Much of the mechanistic research on metformin and DPP4i’s was done before 2014, when the NIH started to promote the study of sex as a biologic variable [47]. However metformin has been found to reduce TNF-alpha, IL-6, and possibly boost IL-10 in females more than males, which is relevant to the pathophysiology of COVID-19 [48–50].

Subgroup analysis was conducted comparing those with a BMI>25kg/m^2^ (the definition of overweight, and the BMI at which visceral adiposity starts to accumulate more rapidly) to those with a BMI<25kg/m^2^ [51]. If metformin were effective only in individuals with an elevated BMI, the antiviral actions of metformin might be less significant than anti-inflammatory and anti-thrombotic effects of metformin. However, we saw no obvious difference between these BMI groups. It is possible that this BMI threshold is too low, or that potential benefit from metformin is not dependent on baseline amount of adipokines (many of which are associated with poor outcomes from COVID-19).

These results may contribute to the growing body of evidence suggesting that metformin use may be associated with less severe COVID-19 disease. There is also in-silico, in-vitro, and in-tissue data suggesting that metformin associated with less severe outcomes from COVID-19 [10, 11, 16–18]. Metformin is safe in nearly all individuals, including individuals with heart, liver, and kidney disease, but should be used with caution in persons with advanced heart, liver, or kidney disease [9, 52–56]. Metformin has very few interactions with other medications and requires no follow-up until after 1 year of use, making it an ideal option for persons on other chronic medications or persons with lack of access to follow-up care.

Given the significant global impact of SARS-CoV-2 and the COVID-19 pandemic, patients should have several options for safe, available, inexpensive early outpatient treatment of SARS-CoV-2 infection to prevent severe COVID-19 disease. There is also evidence that early outpatient treatment with may possibly prevent long COVID symptoms (post-acute sequelae of COVID, PASC) [57].

While in-vitro and in-silico data supports its use in active infection, observational analyses such as this only add information about metformin use before infection with SARS-CoV-2. Few papers describe metformin continued or initiated during hospitalizations for COVID [58]. Randomized trials are needed to understand whether metformin has any efficacy in the setting of SARS-CoV-2 infection, exposure to infection, or treatment and prevention of PASC. Metformin’s safety and cost make it a medication that is low-risk enough to reasonably consider using in a PEP fashion. While viral variants may evade vaccine-induced immunity because of their cell-entry abilities, they will still depend on host proteins for transcription and translation. Metformin’s inhibition of proteins that are critical to viral replication may mean it is still relevant for most viral variants.

### Limitations

This observational analysis is subject to residual unmeasured confounding and bias. The degree of confounding typically seen in the assessment of repurposed medications for outpatient treatment of COVID-19 is not yet well established and in our setting with an active comparator, we would generally assume associations of an unmeasured confounder with treatment to be smaller than associations with the outcome. Because of sample size limitations, we are not able to perform the analysis using a new user active comparator design which may lead to a variety of biases [45]. In order to reduce ascertainment and misclassification bias, analyses were restricted to persons with at least one outpatient healthcare encounter in the previous 12 months, and prescriptions from the previous 90 days [59]. Records of individuals over age 85 were excluded to reduce confounding by frailty, and persons with CKD stages 4, 5, and ESRD were excluded to reduce confounding by contraindication [45]. It is not known whether the persons in these cohorts continued their metformin, SU, and DPP4i use during their SARS-CoV-2 infection. Given that there are several hypotheses as to how metformin might reduce severity of COVID-19 disease, it is not known if use prior to infection, during infection, or after initial acute infection is associated with the results observed in this analysis, and the associations may not generalize beyond adults with type 2 diabetes.

## Conclusions

In this retrospective cohort analysis of adults with T2DM and COVID-19 in a large, geographically diverse dataset there were statistically significant associations between metformin use and less severe outcomes from COVID-19 compared to SU use, but not compared to DPP4i use. Due to the size of the database, this was the first analysis able to compare outcomes across diabetes monotherapy groups, so this manuscript has methodologic strengths over previous observational analyses. This analysis adds to the literature suggesting a potential role for metformin in early treatment and possible post-exposure prophylaxis for COVID-19 disease, but we could not specifically address this hypothesis. Early outpatient treatment with safe and available therapeutics is particularly important for areas of the world with limited access to the vaccines and other COVID-19 therapies. New user cohort studies are needed, but the number of persons initiating oral T2DM treatment during acute SARS-CoV-2 infection may be small. Randomized trials of early outpatient treatment are needed and underway, and randomized trials of post-exposure prophylaxis are also needed.

## Supporting information

S1 File (DOCX)

## References

[1] de Macedo AV. Brazil and COVID-19—A Fleeting Glimpse of What Is to Come. JAMA Health Forum. 2020;1(9):e201061–e. doi: 10.1001/jamahealthforum.2020.1061 36218727

[2] Barbaro M HJT. he Vaccine Trust Problem [Internet]: [Internet]. National Public Radio 2020 July 21, 2020. Podcast.; 2021. Podcast. https://www.nytimes.com/2020/07/21/podcasts/the-daily/coronavirus-vaccine.html

[3] Hildreth JEK, Alcendor DJ. Targeting COVID-19 Vaccine Hesitancy in Minority Populations in the US: Implications for Herd Immunity. Vaccines (Basel). 2021;9(5). doi: 10.3390/vaccines9050489 34064726 PMC8151325

[4] Pushpakom S, Iorio F, Eyers PA, Escott KJ, Hopper S, Wells A, et al. Drug repurposing: progress, challenges and recommendations. Nature Reviews Drug Discovery. 2019;18(1):41–58. doi: 10.1038/nrd.2018.168 30310233

[5] Babar ZU, Ramzan S, El-Dahiyat F, Tachmazidis I, Adebisi A, Hasan SS. The Availability, Pricing, and Affordability of Essential Diabetes Medicines in 17 Low-, Middle-, and High-Income Countries. Front Pharmacol. 2019;10:1375. doi: 10.3389/fphar.2019.01375 31824316 PMC6880243

[6] McDonagh MS, Selph S, Ozpinar A, Foley C. Systematic review of the benefits and risks of metformin in treating obesity in children aged 18 years and younger. JAMA pediatrics. 2014;168(2):178–84. doi: 10.1001/jamapediatrics.2013.4200 24343296

[7] Alqudah A, McKinley MC, McNally R, Graham U, Watson CJ, Lyons TJ, et al. Risk of pre-eclampsia in women taking metformin: a systematic review and meta-analysis. Diabetic Medicine. 2018;35(2):160–72. doi: 10.1111/dme.13523 29044702

[8] Kalafat E, Sukur YE, Abdi A, Thilaganathan B, Khalil A. Metformin for prevention of hypertensive disorders of pregnancy in women with gestational diabetes or obesity: systematic review and meta-analysis of randomized trials. Ultrasound Obstet Gynecol. 2018;52(6):706–14. doi: 10.1002/uog.19084 29749110

[9] Flory J, Lipska K. Metformin in 2019. JAMA. 2019;321(19):1926–7. doi: 10.1001/jama.2019.3805 31009043 PMC7552083

[10] Castle BT, Dock C, Hemmat M, Kline S, Tignanelli C, Rajasingham R, et al. Biophysical modeling of the SARS-CoV-2 viral cycle reveals ideal antiviral targets. bioRxiv. 2020:2020.05.22.111237.

[11] Gordon DE, Jang GM, Bouhaddou M, Xu J, Obernier K, White KM, et al. A SARS-CoV-2 protein interaction map reveals targets for drug repurposing. Nature. 2020;583(7816):459–68. doi: 10.1038/s41586-020-2286-9 32353859 PMC7431030

[12] Singh S, Singh PK, Suhail H, Arumugaswami V, Pellett PE, Giri S, et al. AMP-Activated Protein Kinase Restricts Zika Virus Replication in Endothelial Cells by Potentiating Innate Antiviral Responses and Inhibiting Glycolysis. J Immunol. 2020;204(7):1810–24. doi: 10.4049/jimmunol.1901310 32086387 PMC7310572

[13] Xin G, Wei Z, Ji C, Zheng H, Gu J, Ma L, et al. Metformin Uniquely Prevents Thrombosis by Inhibiting Platelet Activation and mtDNA Release. Sci Rep. 2016;6:36222. doi: 10.1038/srep36222 27805009 PMC5090250

[14] Cameron AR, Morrison VL, Levin D, Mohan M, Forteath C, Beall C, et al. Anti-Inflammatory Effects of Metformin Irrespective of Diabetes Status. Circulation research. 2016;119(5):652–65. doi: 10.1161/CIRCRESAHA.116.308445 27418629 PMC4990459

[15] Ingraham NE, Lotfi-Emran S, Thielen BK, Techar K, Morris RS, Holtan SG, et al. Immunomodulation in COVID-19. Lancet Respir Med. 2020;8(6):544–6. doi: 10.1016/S2213-2600(20)30226-5 32380023 PMC7198187

[16] Schaller MA, Sharma Y, Dupee Z, Nguyen D, Uruena J, Smolchek R, et al. In vitro infection of human lung tissue with SARS-CoV-2: Heterogeneity in host defense and therapeutic response. bioRxiv. 2021:2021.01.20.427541. 10.1172/jci.insight.148003 PMC8492301 34357881

[17] Cariou B, Hadjadj S, Wargny M, Pichelin M, Al-Salameh A, Allix I, et al. Phenotypic characteristics and prognosis of inpatients with COVID-19 and diabetes: the CORONADO study. Diabetologia. 2020;63(8):1500–15. doi: 10.1007/s00125-020-05180-x 32472191 PMC7256180

[18] Crouse A, Grimes T, Peng L, Might M, Ovalle F, Shalev A. METFORMIN USE IS ASSOCIATED WITH REDUCED MORTALITY IN A DIVERSE POPULATION WITH COVID-19 AND DIABETES. 2020. 10.3389/fendo.2020.600439 PMC7838490 33519709

[19] Strollo R, Pozzilli P. DPP4 inhibition: Preventing SARS-CoV-2 infection and/or progression of COVID-19? Diabetes Metab Res Rev. 2020;36(8):e3330. doi: 10.1002/dmrr.3330 32336007 PMC7267128

[20] Solerte SB, Di Sabatino A, Galli M, Fiorina P. Dipeptidyl peptidase-4 (DPP4) inhibition in COVID-19. Acta Diabetol. 2020;57(7):779–83. doi: 10.1007/s00592-020-01539-z 32506195 PMC7275134

[21] Puskarich MA, Cummins NW, Ingraham NE, Wacker DA, Reilkoff RA, Driver BE, et al. A multi-center phase II randomized clinical trial of losartan on symptomatic outpatients with COVID-19. EClinicalMedicine. 2021;37:100957. doi: 10.1016/j.eclinm.2021.100957 34195577 PMC8225661

[22] Luo P, Qiu L, Liu Y, Liu XL, Zheng JL, Xue HY, et al. Metformin Treatment Was Associated with Decreased Mortality in COVID-19 Patients with Diabetes in a Retrospective Analysis. Am J Trop Med Hyg. 2020. doi: 10.4269/ajtmh.20-0375 32446312 PMC7356425

[23] Bramante C, Ingraham N, Murray T, Marmor S, Hoversten S, Gronski J, et al. Observational Study of Metformin and Risk of Mortality in Patients Hospitalized with Covid-19. medRxiv. 2020. doi: 10.1101/2020.06.19.20135095 32607520 PMC7325185

[24] Li Y, Yang X, Yan P, Sun T, Zeng Z, Li S. Metformin in Patients With COVID-19: A Systematic Review and Meta-Analysis. Frontiers in Medicine. 2021;8. doi: 10.3389/fmed.2021.704666 34490296 PMC8416892

[25] Haendel MA, Chute CG, Bennett TD, Eichmann DA, Guinney J, Kibbe WA, et al. The National COVID Cohort Collaborative (N3C): Rationale, design, infrastructure, and deployment. Journal of the American Medical Informatics Association. 2020;28(3):427–43. 10.1093/jamia/ocaa196 PMC7454687 32805036

[26] Health MDo. [https://www.health.state.mn.us/diseases/coronavirus/stats/.

[27] Pogue J, Thabane L, Devereaux PJ, Yusuf S. Testing for heterogeneity among the components of a binary composite outcome in a clinical trial. BMC Medical Research Methodology. 2010;10(1):49. doi: 10.1186/1471-2288-10-49 20529275 PMC2909251

[28] Lipsitch M, Tchetgen Tchetgen E, Cohen T. Negative controls: a tool for detecting confounding and bias in observational studies. Epidemiology (Cambridge, Mass). 2010;21(3):383–8. doi: 10.1097/EDE.0b013e3181d61eeb 20335814 PMC3053408

[29] Levey AS, Stevens LA. Estimating GFR Using the CKD Epidemiology Collaboration (CKD-EPI) Creatinine Equation: More Accurate GFR Estimates, Lower CKD Prevalence Estimates, and Better Risk Predictions. American Journal of Kidney Diseases. 2010;55(4):622–7. doi: 10.1053/j.ajkd.2010.02.337 20338463 PMC2846308

[30] Rubin D. Multiple Imputation for Nonresponse in Surveys. Statistical Papers 1990(1):180-.

[31] Hainmueller J. Entropy Balancing for Causal Effects: A Multivariate Reweighting Method to Produce Balanced Samples in Observational Studies. Political Analysis. 2012;20(1):25–46.

[32] Chan KC, Yam SC, Zhang Z. Globally efficient non-parametric inference of average treatment effects by empirical balancing calibration weighting. J R Stat Soc Series B Stat Methodol. 2016;78(3):673–700. doi: 10.1111/rssb.12129 27346982 PMC4915747

[33] Zhao Q, Percival D. Entropy Balancing is Doubly Robust. Journal of Causal Inference. 2017;5(1).

[34] Chattopadhyay A, Hase CH, Zubizarreta JR. Balancing vs modeling approaches to weighting in practice. Stat Med. 2020;39(24):3227–54. doi: 10.1002/sim.8659 32882755

[35] Funk MJ, Westreich D, Wiesen C, Stürmer T, Brookhart MA, Davidian M. Doubly robust estimation of causal effects. Am J Epidemiol. 2011;173(7):761–7. doi: 10.1093/aje/kwq439 21385832 PMC3070495

[36] VanderWeele TJ, Ding P. Sensitivity Analysis in Observational Research: Introducing the E-Value. Annals of internal medicine. 2017;167(4):268–74. doi: 10.7326/M16-2607 28693043

[37] ROSENBAUM PR. Sensitivity analysis for certain permutation inferences in matched observational studies. Biometrika. 1987;74(1):13–26.

[38] van Buuren S, Groothuis-Oudshoorn K. mice: Multivariate Imputation by Chained Equations in R. Journal of Statistical Software. 2011;45(3):1–67.

[39] Computing RFfS. Weight It.

[40] Pishgar F, Greifer N, Leyrat C, Stuart E. MatchThem:: Matching and Weighting after Multiple Imputation

[41] Computing RFfS. cobalt: Covariate Balance Tables and Plots.

[42] Computing RFfS. survey: Analysis of Complex Survey Samples.

[43] Computing RFfS. tableone: Create “Table 1” to Describe Baseline Characteristics with or without Protensity Score Weights.

[44] Wasserstein RL, Lazar NA. The ASA Statement on p-Values: Context, Process, and Purpose. The American Statistician. 2016;70(2):129–33.

[45] Wang J, Cooper JM, Gokhale K, Acosta-Mena D, Dhalla S, Byne N, et al. Association of Metformin with Susceptibility to COVID-19 in People with Type 2 Diabetes. The Journal of Clinical Endocrinology & Metabolism. 2021;106(5):1255–68. doi: 10.1210/clinem/dgab067 33560344 PMC7928949

[46] Diabetes Prevention Program Research G. Lipid, Lipoproteins, C-Reactive Protein, and Hemostatic Factors at Baseline in the Diabetes Prevention Program. Diabetes care. 2005;28(10):2472–9. doi: 10.2337/diacare.28.10.2472 16186282 PMC1404506

[47] Clayton JA, Collins FS. Policy: NIH to balance sex in cell and animal studies. Nature. 2014;509(7500):282–3. doi: 10.1038/509282a 24834516 PMC5101948

[48] Matsiukevich D, Piraino G, Lahni P, Hake PW, Wolfe V, O’Connor M, et al. Metformin ameliorates gender-and age-dependent hemodynamic instability and myocardial injury in murine hemorrhagic shock. Biochimica et Biophysica Acta (BBA)—Molecular Basis of Disease. 2017;1863(10, Part B):2680–91. doi: 10.1016/j.bbadis.2017.05.027 28579457 PMC5653443

[49] Quan H, Zhang H, Wei W, Fang T. Gender-related different effects of a combined therapy of Exenatide and Metformin on overweight or obesity patients with type 2 diabetes mellitus. J Diabetes Complications. 2016;30(4):686–92. doi: 10.1016/j.jdiacomp.2016.01.013 26873871

[50] Park JW, Lee JH, Park YH, Park SJ, Cheon JH, Kim WH, et al. Sex-dependent difference in the effect of metformin on colorectal cancer-specific mortality of diabetic colorectal cancer patients. World J Gastroenterol. 2017;23(28):5196–205. doi: 10.3748/wjg.v23.i28.5196 28811714 PMC5537186

[51] Bosch TA, Dengel DR, Kelly AS, Sinaiko AR, Moran A, Steinberger J. Visceral adipose tissue measured by DXA correlates with measurement by CT and is associated with cardiometabolic risk factors in children. Pediatric obesity. 2015;10(3):172–9. doi: 10.1111/ijpo.249 24990328 PMC5927585

[52] Bailey CJ. Metformin: historical overview. Diabetologia. 2017;60(9):1566–76. doi: 10.1007/s00125-017-4318-z 28776081

[53] Lipska KJ, Krumholz H, Soones T, Lee SJ. Polypharmacy in the Aging Patient: A Review of Glycemic Control in Older Adults With Type 2 Diabetes. JAMA. 2016;315(10):1034–45. 26954412 10.1001/jama.2016.0299 PMC4823136

[54] Iranshahy M, Rezaee R, Karimi G. Hepatoprotective activity of metformin: A new mission for an old drug? Eur J Pharmacol. 2019;850:1–7. doi: 10.1016/j.ejphar.2019.02.004 30753869

[55] de la Cuesta-Zuluaga J, Mueller NT, Corrales-Agudelo V, Velásquez-Mejía EP, Carmona JA, Abad JM, et al. Metformin Is Associated With Higher Relative Abundance of Mucin-Degrading Akkermansia muciniphila and Several Short-Chain Fatty Acid-Producing Microbiota in the Gut. Diabetes care. 2017;40(1):54–62. doi: 10.2337/dc16-1324 27999002

[56] Salpeter SR, Greyber E, Pasternak GA, Salpeter Posthumous EE. Risk of fatal and nonfatal lactic acidosis with metformin use in type 2 diabetes mellitus. Cochrane Database Syst Rev. 2010(1):Cd002967. doi: 10.1002/14651858.CD002967.pub3 20393934 PMC7138050

[57] Seftel D, Boulware DR. Prospective Cohort of Fluvoxamine for Early Treatment of Coronavirus Disease 19. Open Forum Infect Dis. 2021;8(2):ofab050. doi: 10.1093/ofid/ofab050 33623808 PMC7888564

[58] Cheng X, Liu Y-M, Li H, Zhang X, Lei F, Qin J-J, et al. Metformin Is Associated with Higher Incidence of Acidosis, but Not Mortality, in Individuals with COVID-19 and Pre-existing Type 2 Diabetes. Cell Metabolism. 2020;32(4):537–47.e3. 32861268 10.1016/j.cmet.2020.08.013 PMC7439986

[59] Joshua Lin K, Jin Y, Gagne J, Glynn RJ, Murphy SN, Tong A, et al. Longitudinal Data Discontinuity in Electronic Health Records and Consequences for Medication Effectiveness Studies. Clin Pharmacol Ther. 2022;111(1):243–51. doi: 10.1002/cpt.2400 34424534 PMC8678205

