# Supplementary material for: Diabetes medications and associations with Covid-19 outcomes in the N3C database: A national retrospective cohort study
Source: PLoS One. 2022 Nov 17;17(11):e0271574. doi: 10.1371/journal.pone.0271574 (PMC9671347; doi:10.1371/journal.pone.0271574)
Supplement: S1 File — (DOCX) [file pone.0271574.s001.docx]

| **Supplemental Table. Risk Ratios for severe COVID-19 outcomes in subgroups by body mass index (BMI).** | | | | | | |
| --- | --- | --- | --- | --- | --- | --- |
| **Metformin versus DPP4i** | | **BMI < 25kg/m^2^** | | | **BMI** $\boldsymbol{\geq}$ **25kg/m^2^** | |
| **Outcome** | **Model** | **RR (95% CI)** | **p value** | | **RR (95% CI)** | **p value** |
| Hospitalization | Crude | 0.68 (0.43-1.08) | 0.11 | | 0.93 (0.73-1.20) | 0.59 |
|  | Adjusted | 0.59 (0.36-0.97) | 0.04 | | 1.01 (0.72-1.40) | 0.97 |
| Ventilation | Crude | 0.19 (0.04-0.84) | 0.03 | | 0.57 (0.29-1.14) | 0.11 |
|  | Adjusted | 0.43 (0.09-2.13) | 0.30 | | 0.64 (0.29-1.45) | 0.29 |
| Mortality | Crude | 0.40 (0.15-1.08) | 0.07 | | 0.67 (0.32-1.39) | 0.28 |
|  | Adjusted | 0.42 (0.18-1.01) | 0.05 | | 0.93 (0.40-2.16) | 0.86 |
| Back pain  (negative control) | Crude | 1.30 (0.72-2.34) | 0.39 | | 0.91 (0.77-1.07) | 0.27 |
|  | Adjusted | 2.09 (0.88-4.94) | 0.09 | | 0.95 (0.77-1.16) | 0.60 |
| **Metformin versus SU** | | **BMI < 25kg/m^2^** | | | **BMI** $\boldsymbol{\geq}$ **25kg/m^2^** | |
| **Outcome** | **Model** | **RR (95% CI)** | | **p value** | **RR (95% CI)** | **p value** |
| Hospitalization | Crude | 0.80 (0.52-1.23) | | 0.31 | 0.93 (0.78-1.11) | 0.40 |
|  | Adjusted | 1.07 (0.66-1.73) | | 0.78 | 0.98 (0.81-1.20) | 0.87 |
| Ventilation | Crude | 0.28 (0.06-1.23) | | 0.09 | 0.64 (0.38-1.09) | 0.10 |
|  | Adjusted | 0.39 (0.07-2.13) | | 0.28 | 0.51 (0.26-0.99) | 0.05 |
| Mortality | Crude | 0.59 (0.22-1.59) | | 0.30 | 0.47 (0.30-0.75) | <0.01 |
|  | Adjusted | 0.70 (0.24-2.03) | | 0.51 | 0.52 (0.29-0.93) | 0.03 |
| Back pain  (negative control) | Crude | 0.86 (0.58-1.29) | | 0.47 | 1.00 (0.88-1.14) | 0.97 |
|  | Adjusted | 1.13 (0.67-1.89) | | 0.65 | 1.01 (0.88-1.17) | 0.86 |

Abbreviations: BMI=body mass index. DPP4i=dipeptidyl peptidase-4 inhibitors; SU=sulfonylureas.

**Supplemental Figure 1:** The x axis the standardized mean difference for continuous variables and difference in proportions for categorical variables for the 100 terms with the greatest imbalance prior to weighing. The circles represent the balance before weighting and the squares represent the balance after weighing.

**Supplemental Figure 2:** Sensitivity analysis using larger windows for medication use.

**Supplemental Figure 2.** This represents a sensitivity analysis of the data presented in Figure 3 of the main manuscript. This analysis uses a filter of 180-days and 270 days as opposed to 90 days. The risk ratios and 95% confidence intervals for the overall cohort. The two panels on the left represent the metformin vs. DPP4i inhibitor comparison. The two panels on the right represent the metformin vs. SU comparison. The circles represent the raw comparison, and the squares represent the adjusted analysis. Within each panel, from top to bottom, the top result is risk of hospitalization; the 2^nd^ result is risk of ventilation (including ECMO); the 3^rd^ result is the risk of mortality; and the 4^th^ comparison is the risk of a back pain, calculated as a negative control outcome. Abbreviations: MET=metformin; DPP4i=dipeptidyl peptidase 4 inhibitors; SU=sulfonylurea.
