# Supplementary material for: Diabetes medications and associations with Covid-19 outcomes in the N3C database: A national retrospective cohort study
Source: PLoS One. 2022 Nov 17;17(11):e0271574. doi: 10.1371/journal.pone.0271574 (PMC9671347; doi:10.1371/journal.pone.0271574)
Supplement: Attachment — Submitted filename: Plos.Response.April.docx [file pone.0271574.s002.docx]

PONE-D-21-35574R1
Diabetes medications and Associations with Covid-19 Outcomes in the N3C Database: A National Retrospective Cohort Study
Dr. Carolyn T Bramante

Dear Dr. Bramante,

We've checked your submission and before we can proceed, we need you to address the following issues:

1. Please include a legend for figure 4

*I apologize, there is no figure 4. That was a typo within the manuscript. I have updated that on the Tracked and Clean versions.*

2. We note that the grant information you provided in the ‘Funding Information’ and ‘Financial Disclosure’ sections do not match.

When you resubmit, please ensure that you provide the updated Funding Information.

*In March, you sent us this clarification and we sent this response. I think you are saying you want me to move information from the “Financial Disclosure” section to “Funding Information.” We have published in other journals with the information as submitted, but I have made that addition here. You said that you would update it online for us previously, let me know if that is not the case.*

The funding statement is below:

“The analyses described in this publication were conducted with data or tools accessed through the National Institutes of Health’s National Center for Advancing Translational Sciences (NCATS) N3C Data Enclave (<https://covid.cd2h.org>) and N3C Attribution & Publication Policy v 1.2-2020-08-25b supported by NCATS U24 TR002306 and NCATS grants KL2TR002492 (UMN) and UL1TR002494 (UMN) and UL1TR002489 (UNC); and the National Institute of Digestive, Diabetes, and Kidney diseases K23DK124654 (CB), and K23DK116935 (JT). The content is solely the responsibility of the authors and does not necessarily represent the official views of the National Institutes of Health’s National Center for Advancing Translational Sciences.

JBB is supported by grants from the National Institutes of Health, Patient Centered Outcomes Research Institute, Juvenile Diabetes Research Foundation International and the American Diabetes Association. he reports grant support from AstraZeneca, Dexcom, Eli Lilly, Intarcia Therapeutics, Johnson & Johnson, Lexicon, NovaTarg, Novo Nordisk, Sanofi, Theracos, Tolerion, and vTv Therapeutics; he has received fees for consultation from Anji Pharmaceuticals, AstraZeneca, Boehringer Ingelheim, Cirius Therapeutics Inc, Eli Lilly, Fortress Biotech, Janssen, Mellitus Health, Moderna, Pendulum Therapeutics, Praetego, Stability Health, and Zealand Pharma;

TS receives investigator-initiated research funding and support as Principal Investigator (R01 AG056479) from the National Institute on Aging (NIA), and as Co-Investigator (R01 HL118255, R01MD011680), National Institutes of Health (NIH). He also receives salary support as Director of Comparative Effectiveness Research (CER), NC TraCS Institute, UNC Clinical and Translational Science Award (UL1TR002489), the Center for Pharmacoepidemiology (current members: GlaxoSmithKline, UCB BioSciences, Takeda, AbbVie, Boehringer Ingelheim), from pharmaceutical companies (Novo Nordisk), and from a generous contribution from Dr. Nancy A. Dreyer to the Department of Epidemiology, University of North Carolina at Chapel Hill. Dr. Stürmer does not accept personal compensation of any kind from any pharmaceutical company. He owns stock in Novartis, Roche, and Novo Nordisk.

The funders had no role in study design, data collection and analysis, decision to publish, or preparation of the manuscript.”

3. We note your current Data Availability statement is:

- "Yes - all data are fully available without restriction"
- "The data is available in the N3C public repository. Individuals must do training to be cleared in order to access the repository."

We note that you have indicated that data from this study are available upon request. PLOS only allows data to be available upon request if there are legal or ethical restrictions on sharing data publicly. For more information on unacceptable data access restrictions, please see <http://journals.plos.org/plosone/s/data-availability#loc-unacceptable-data-access-restrictions>.

Before we proceed with your manuscript, please address the following prompts:

a) If there are no restrictions, please upload the minimal anonymized data set necessary to replicate your study findings to a stable, public repository and provide us with the relevant URLs, DOIs, or accession numbers. For a list of recommended repositories, please see <https://journals.plos.org/plosone/s/recommended-repositories>. You also have the option of uploading the data as Supporting Information files, but we would recommend depositing data directly to a data repository if possible.

b) If there are ethical or legal restrictions on sharing a de-identified data set, please explain them in detail (e.g., data contain potentially identifying or sensitive patient information, data are owned by a third-party organization, etc.) and who has imposed them (e.g., a Research Ethics Committee or Institutional Review Board, etc.). Please also non-author provide contact information* for a data access committee, ethics committee, or other institutional body to which data requests may be sent.

*PLOS’ Data Policy states that authors cannot be the sole named individuals responsible for ensuring data access (<http://journals.plos.org/plosone/s/data-availability#loc-acceptable-data-sharing-methods>).

*There are ethical restrictions on sharing a de-identified data set because the data contain potentially identifying or sensitive patient information. A Research Ethics and Data Download Committee have imposed these restrictions, not any of the authors on this manuscript. The data are available in the N3C public repository at no cost to anyone who does on-line training that is required in order to access the repository.*

We will update your Data Availability statement on your behalf to reflect the information you provide.

We've returned your manuscript to your account. Please resolve these issues and resubmit your manuscript within 21 days. If you need more time, please email the journal office at [plosone@plos.org](mailto:plosone@plos.org). We are happy to grant extensions of up to one month past this due date. If we do not hear from you within 21 days, we will withdraw your manuscript.

Please log on to PLOS Editorial Manager at <https://www.editorialmanager.com/pone/> to access your manuscript. You will find your manuscript in the 'Submissions Sent Back to Author' link under the New Submissions menu. Be sure to remove your previous manuscript file if you are uploading a new file in response to these requests. After you've made the changes requested above, please be sure to view and approve the revised PDF after rebuilding the PDF to complete the resubmission process.

We are requesting these changes to comply with the PLOS ONE submission guidelines (<https://journals.plos.org/plosone/s/submission-guidelines>). Please note that we won't send your manuscript for review until you have resolved the above requests. 

Thank you for submitting your work to PLOS ONE and supporting our mission of Open Science.


Kind regards,

Rose Ann Joyce Sagun Puetes
PLOS ONE
